# Cost and cost-effectiveness of community based and health facility based directly observed treatment of tuberculosis in Dar es Salaam, Tanzania

**DOI:** 10.1186/1478-7547-3-6

**Published:** 2005-07-14

**Authors:** Eliud Wandwalo, Bjarne Robberstad, Odd Morkve

**Affiliations:** 1 Centre for International Health, University of Bergen, Armauer Hansen Building, N-5021, Bergen, Norway; 2 National Tuberculosis and Leprosy Programme, Ministry of Health, P.O Box 9083, Dar es Salaam, Tanzania

## Abstract

**Background:**

Identifying new approaches to tuberculosis treatment that are effective and put less demand to meagre health resources is important. One such approach is community based direct observed treatment (DOT). The purpose of the study was to determine the cost and cost effectiveness of health facility and community based directly observed treatment of tuberculosis in an urban setting in Tanzania.

**Methods:**

Two alternative strategies were compared: health facility based directly observed treatment by health personnel and community based directly observed treatment by treatment supervisors. Costs were analysed from the perspective of health services, patients and community in the year 2002 in US $ using standard methods. Treatment outcomes were obtained from a randomised-controlled trial which was conducted alongside the cost study. Smear positive, smear negative and extra-pulmonary TB patients were included. Cost-effectiveness was calculated as the cost per patient successfully treated.

**Results:**

The total cost of treating a patient with conventional health facility based DOT and community based DOT were $ 145 and $ 94 respectively. Community based DOT reduced cost by 35%. Cost fell by 27% for health services and 72% for patients. When smear positive and smear negative patients were considered separately, community DOT was associated with 45% and 19% reduction of the costs respectively. Patients used about $ 43 to follow their medication to health facility which is equivalent to their monthly income. Indirect costs were as important as direct costs, contributing to about 49% of the total patient's cost. The main reason for reduced cost was fewer number of visits to the TB clinic. Community based DOT was more cost-effective at $ 128 per patient successfully treated compared to $ 203 for a patient successfully treated with health facility based DOT.

**Conclusion:**

Community based DOT presents an economically attractive option to complement health facility based DOT. This is particularly important in settings where TB clinics are working beyond capacity under limited resources.

## Background

Tuberculosis (TB) is among the top ten causes of global mortality and morbidity accounting for about 26% of all preventable deaths [1,2]. In Tanzania, more than 60,000 new TB patients are notified annually and TB is the third leading killer of adults behind Malaria and Acquired Immune-deficiency syndrome (AIDS) [3,4]. The rapid annual increase of TB cases in the country is largely attributed to Human immunodeficiency Virus (HIV).

The importance of TB/HIV co-infection as a major public health problem in Tanzania is not only due to its high incidence and mortality but also to its social and economic consequences. Tanzania is one of the several countries experiencing a reversal in human development mainly due HIV/AIDS and TB epidemics [5]. The majority of people with TB/HIV co infection are in the economic productive age group and the World Bank estimates that the gross domestic product (GDP) will be 15–20% lower in 2015 than it would have been without the AIDS pandemic [3,5].

The internationally recommended DOTS (Directly Observed Treatment-short course) strategy has been implemented in the country for more than 18 years mainly in public health facilities with treatment directly observed by trained health staff. However, fewer than half of all health facilities in the country provide TB services. Efforts to expand DOTS to more health facilities have been limited by scarce resources and understaffing. This leads to overcrowding of patients especially in urban settings like Dar es Salaam, which notify about a quarter of all TB patients in the country. The rising number of TB cases and critical shortage of skilled staff has put considerable strain on the public health system. With a public health budget of about US $ 6 per capita per year, the TB epidemic significantly influences the Tanzanian health system performance. Therefore, identifying new approaches to treatment that maintain effectiveness and put less demands to the meagre health resources by reducing pressure and lowering cost of delivering TB treatment becomes a priority. One such approach is community based direct observed treatment (DOT).

Supervision of TB patients in the community has been piloted in a variety of settings with successful results [6-9]. However, little is known about the cost and cost effectiveness of this option in Tanzania. The economic analysis is important to assist policy makers in decision making and rational allocation of scarce health resources. We conducted the study to determine the cost and cost effectiveness of health facility and community based DOT in an urban setting in Tanzania.

## Methods

### Study setting

The study was conducted in Temeke district in Dar es Salaam city. Temeke is an urban district with low socio-economic indicators characterised by rapid population growth and overburdened public health services. The district has a population of about 700,000 people with a health facility to population ratio of about 1 per 5000. Tuberculosis and AIDS are leading causes of death in the district, representing 36.5% of years of life lost for all age groups [10].

### Alternative strategies

Two treatment options were compared; health facility based DOT option and community based DOT option using treatment supervisors. A brief description of the alternatives is given below, as full details are available elsewhere [6].

The conventional (health facility based DOT) approach to tuberculosis control in Tanzania presupposes initial diagnosis and treatment of patients on ambulatory basis, provided the patients are strong enough to follow their medication and are free from complications that need hospital care[11]. During the intensive two months period, patients visit health facility daily for direct observation of treatment by trained health personnel. This is followed by a 6-month continuation phase period where patients visit a health facility once per month. Before 2003 and during the study period, smear negative and extra pulmonary TB patients received intermittent drug regimen three times weekly. Currently all patients receive daily treatment

In the new treatment option (community based DOT) direct observation of treatment was supervised by treatment supervisors rather than health facility personnel as in the conventional treatment. The supervisors were guardians and former TB patients. A guardian was defined as a family member or a close relative living with patient. Patients were assisted in the selection of a responsible, trusted guardian. Willing former TB patients who had successfully completed treatment and lived close to the patients' home were selected by the district tuberculosis and leprosy co-ordinators (DTLC). Treatment supervisors collected drugs from health facilities once per week during the entire two months intensive period. During this period a health worker also made surprise visits to patients' homes to check for adherence of treatment by reviewing treatment cards and pill count. Patients were also requested to report to the TB clinics fortnightly for clinical evaluation and monitoring of their progress. After the first two months, treatment was exactly as in the conventional health facility option.

### Study participants

The study was conducted alongside a randomised-controlled study [6]. To select patients to be included in this study, a systematic sampling method was used. A list of all patients who participated in the randomised trial was drawn from the TB registers. Patients were listed consecutively as they appeared in the TB register. Separate lists were prepared for patients who received community DOT and health facility DOT. From the lists every fifth patient was picked to participate in the present study. The corresponding supervisors of the patients allocated to community DOT were also interviewed. The study population consisted of tuberculosis patients over 5 years old who started treatment in the district. New smear positive and smear negative pulmonary tuberculosis as well as extra pulmonary tuberculosis patients were included in the study. Patients were excluded from the study, if they had been previously treated for TB and if they had severe illness that precluded ambulatory treatment. Patients were recruited from five diagnostic centres in the district with high caseload (more than 100 patients per year). The study was conducted between December, 2001 and January 2003.

### Costing

Costs were assessed from a societal perspective as advocated by current standards for cost-effectiveness analysis [12-14]. Average costs of each component of care and treatment was calculated as the quantity of resources used multiplied with their unit price. Costs associated with diagnosis were not included in the analysis, since approaches to diagnosis were the same for all options. Furthermore, costs associated with TB diagnosis would have been difficult to accurately establish because of the long delays in diagnosis and the fact that these costs were incurred before TB was confirmed. The exclusion of the cost of diagnosis in the study does not however influence the relative ranking of community DOT and health facility based DOT, but means that comparison with other health interventions such as malaria control cannot be done.

Broadly, the costs were categorised as "provider" and "personal" or "community". Provider cost is associated with developing and operating a health care service. They include staff costs, supplies and equipment. Provider's costs were incurred by the health system, tuberculosis programme and the community TB project. Personal or community costs are those incurred by patients and treatment supervisors. They include direct and indirect costs. Direct costs are non-medical costs related to visiting the TB clinic such as transport costs, buying food and drinks when visiting a TB clinic. Direct medical costs such as drug costs are included as provider's costs because they were incurred by the TB programme. Indirect costs refer to the value of lost time by the patients and treatment supervisors to follow up TB treatment. This should not be confused with overheads of fixed costs used in accounting practices as pointed by some authors [12].

Costs of a visit to a TB clinic were calculated from one health facility (Mbagala) in Temeke. Joint costs (costs items which were used for more than one activity) were allocated according to the proportion of the time the cost item was used for TB activity. Capital costs were annualised using a discount rate of 10%. This has been used in other studies in Tanzania and has been recommended by the Bank of Tanzania in 2002 [15,16]. Annualisation was done on the assumption that the expected useful life of buildings was 30 years, for vehicles and equipment 10 years and for motorcycles 5 years. The base year for valuing costs was 2002, and exchange rate applicable at that time was 967 Tanzanian Shillings to US $1.

Sources of data included budget and expenditure files for Temeke district, health facilities, the community TB project in Temeke and the National Tuberculosis and Leprosy Programme (NTLP). Others include salary scales for established positions, reports from Temeke municipal, Ministry of Health and the Bank of Tanzania. Face to face interviews were conducted with the staff of the health facilities, Temeke Municipal and Ministry of Health.

Patients costs were estimated using a structured questionnaire. Patients were interviewed about travel and time costs associated with a visit to the TB clinic, their average monthly income and other costs associated with TB treatment and care. Time costs were converted to a monetary value based on the average reported income among interviewed patients (including all sources of incomes). Costs incurred by the treatment supervisors were assessed by a structured questionnaire by asking about the time and travel costs to collect drugs as well as supervising a patient to take medication, and other costs (incurred) including training and supervision of community DOT. Costs per patients treated in different treatment options (community VS health facility) were calculated as a weighted average of the costs according to TB type (smear positive, smear negative and extrapulmonary)

### Effectiveness

Measure of effectiveness used was treatment success. Data on treatment outcomes were obtained from the randomised-controlled trial and operational definitions used in this study are explained in detail elsewhere [6]. Briefly, treatment success included patients who were cured and those who completed treatment. Cured patients were those with positive sputum smear before starting treatment and confirmed to be sputum negative at 7(or 8) months and at least one previous occasion. Completed treatment applied to: patients who had positive pre-treatment results, negative results at 2 months, and no end of treatment results; patients who had negative pre-treatment results and had been placed on treatment for clinical reasons, and patients who completed the full course of treatment, but had no pre-treatment or end-of-treatment bacteriological results.

The term treatment success is used in routine practice to refer to smear positive patients who are cured and have completed treatment. It is worth noting that we have expanded this definition to include smear negative and extra pulmonary tuberculosis patients who completed treatment as well, since our aim is to compare alternative treatment strategies regardless of the patient's TB type. This operational definition has also been used by other researchers [17].

Estimates of cure rate that would apply without treatment (null intervention scenario) was calculated assuming a self cure rate of 20% in the absence of treatment for individuals who are not otherwise immuno-compromised, and a self cure rate of 0% for those who are HIV+[13,18]. This can be calculated from the formula: {(estimated percentage of patients who are HIV + x 0) + (estimated percentage of patients who are HIV- x 20) }/100

### Cost-effectiveness

Cost effectiveness was calculated as the average cost per patient treated successfully. This was done by dividing the total cost and patients successfully treated.

### Sensitivity analysis

One way sensitivity analyses were undertaken to assess the robustness of the results to changes in key input variables. The uncertainty analyses were based on most likely, minimum and maximum values. For valuing time cost, average reported income of the patients was used as the base-case estimates. Sensitivity analysis was conducted when time was valued as zero for all patients and when time was valued for patients with income only. For the effectiveness data, lower and upper boundaries of 95% confidence interval of the treatment outcome was used. Sensitivity analysis was undertaken with and without 'no treatment 'option (Table 3)

**Table 3:** Influence of changes in key input variables on cost-effectiveness ratio in US $ per patient successful treated

Input variable	Input value		Average CER community DOT^a^		Average CER health facility DOT^a^	
	Min.	Max.	Min.	Max.	Min.	Max.

Patient time loss^b^	0	US$0.29	117	130	168	203
Supporter time loss^b^	0	US $ 0.48	123	129	196	196
Discount rate	0	3%	125	125	186	188
Success rate(community DOT)^c^	69%	78%	136	120		
Success rate (health facility DOT)^c^	62%	74%			281	235
Success rate(community DOT)^d^	81%	89%	116	106		
Success rate (health facility DOT)^d^	79%	87%			184	167

^a^The sensitivity analyses are based on base-case calculations

^b^time loss: minimum=assuming time is valued as zero; maximum= average income per hour among those with income only

^c^Success rate taking in consideration cure rate in 'no treatment' scenario

^d^Success rate without cure rate in 'no treatment' scenario

## Results

### Study participants

A total of 103 TB patients and 42 treatment supervisors were enrolled in the study. Among the patients 45 (44%) received DOT in the community and 58 (56%) received DOT in health facility. The majority of the patients were male 63 (61%) and the mean age of the patients was 32 years (median 30). Most of the patients 67 (65%) completed primary education and worked in informal sector or self-employed 43 (42%). The average reported income of the patients was USD 43. No significant difference (p > 0.05) was found between patients treated in community and health facilities on social-demographic variables (Table 1). The majority of the treatment supervisors were guardians (39, 91%) and females (27, 63%). Their mean age was 37 years (SD 12, median 38).

**Table 1:** General characteristics of the patients

	**Community DOT n (%)**	**Health facility DOT n (%)**
**Sex**		
Male	26 (57.8)	37 (63.8)
Female	19 (42.2)	21 (36.2)
**Age (in years)**		
Mean (SD)	31 (9)	33 (12)
**Education status**		
No education	5 (11.1)	4 (6.9)
Not completed primary school	9 (20.0)	7 (12.1)
Completed primary school	25 (55.6)	42(72.4)
Secondary school and above	6 (13.3)	4 (6.9)
Others	0	1(1.7)
**Occupation**		
Informal and self employed	21 (46.7)	22 (37.9)
Employed	14 (31.1)	14 (24.1)
No employment	5 (11.1)	7 (12.1)
Housewife	5 (11.1)	15 (25.9)
**Home-TB clinic distance**		
Below 1 km	29 (64.4)	46 (79.3)
1–2 kms	10 (22.2)	7 (12.1)
Above 2 kms	6 (13.3)	5 (8.6)
TB type		
Smear- positive PTB	25 (55.6)	33 (56.9)
Smear- negative PTB and extra-pulmonary TB	20 (44.4)	25 (43.1)

### Costs

**Figure 1:**
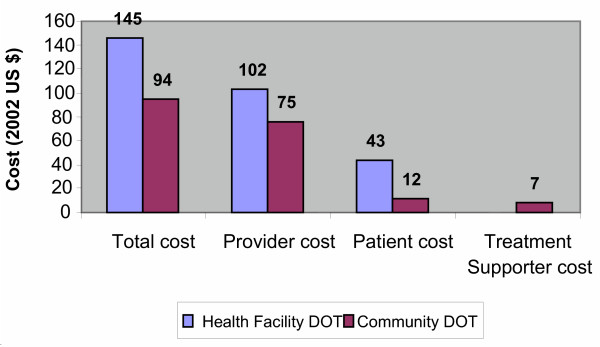
Average cost per patient (all patients).

The average cost of treating patients under the alternative DOT options is shown in figure 1. Overall, treating a patient under community DOT was less costly by 35% compared to health facility based DOT. The total cost of treating a patient under health facility DOT was US 145 compared to USD 94 for treating a patient under community DOT. Costs were reduced from all perspectives: providers (TB programme and community project) and community (patients and treatment supervisors). Community DOT reduced providers cost by 27%, patients cost by 72%, and combined patients and treatment supporter cost (community cost) by 55%.

Providers cost was about 70% (USD 102) of the total health facility DOT cost compared to 80 %(USD 75) of community DOT total cost (Table 2). A visit to the TB clinic was the main cost item of health facility DOT, constituting about 55% (USD 56) of the providers cost. This cost item included all recurrent and capital clinic costs excluding drugs and investigations. Overall management and supervision was the main cost item under community based DOT constituting to about 53% (USD 40) of providers cost (Table 2).

**Table 2:** Average cost per patient for different items of alternative DOT options

	Health facility DOT					Community DOT				
**Provider's cost**	Smear positive		Smear negative&EPTB		Total health facility USD*	Smear positive		Smear negative&EPTB		Total community USD*

	quantity	Unit price USD	quantity	Unit price USD		quantity	Unit price USD	quantity	Unit price USD	

Visit TB clinic	58	1.12	24	1.90	***56.0***	10	1.12	10	1.90	***15.0***
Drugs	1	20.90	1	12.10	***17.0***	1	20.90	1	12.10	***17.0***
NTLP management & supervision district level	1	1.50	1	1.50	***1.5***	1	1.50	1	1.50	***1.50***
NTLP management & supervision regional level	1	0.93	1	0.93	***0.9***	1	0.93	1	0.93	***0.90***
Community project management	1	26.97	1	26.97	***27.0***	1	26.97	1	26.97	***27.0***
Community project supervision						1	13.16	1	13.16	***13.0***
Community project training						1	0.90	1	0.90	***0.9***
*Total providers cost*					***102***					***75***
**Patient's cost**										
Direct	58	0.51	24	0.51	***22.0***	10	0.51	10	0.51	***5.0***
Indirect	58	0.50	24	0.51	***21.0***	1	7.54	1	5.90	***7.0***
*Total patient's cost*					***43***					***12***
**Treatment supporter's cost**										
Direct						8	0.24	8	0.24	***1.92***
Indirect						1	6.18	1	4.54	***5.43***
*Total treatment supporter's cost*										***7***
***Total cost***					***145***					***94***

*Weighted average of smear positives(56%) and smear negatives and extra- pulmonary (44%)

Patients costs was about 30% (USD 43) of all health facility DOT cost compared 13 % (USD 12) of community DOT cost. The combined patients and treatment supervisors cost (community cost) was 20 %(USD 19) of all cost under the community DOT option (Figure 1 and Table 2). Indirect patients cost under health facility DOT was as important as direct cost, accounting for about 49% of patients' cost. Indirect cost was attributed mainly to the time lost from work. Patients lost an average of 2 hours daily to follow up TB treatment in the health facility.

**Figure 2:**
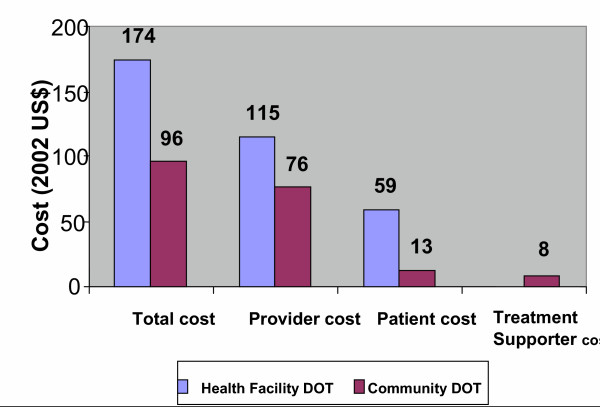
Average cost per patient (smear positive).

**Figure 3:**
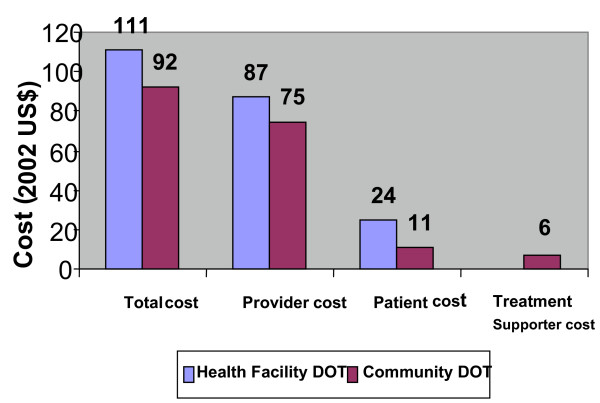
Average cost per patient (smear negative and extra pulmonary).

Separate analysis of the cost according to TB type, showed a marked reduction in total cost among smear positive patients treated under community DOT compared to smear negative and extra pulmonary treated under the same option. Cost fell by 45% and 19% respectively (Figures 2 and 3).

### Effectiveness

A total of 587 patients were recruited to study the effectiveness of TB treatment. Among them 260 were randomised to community based DOT and 327 to health facility based DOT. Both DOT options gave similar treatment outcomes. Treatment success rates among patients under community and health facility based DOT were 85 %(221 patients) and 83 %(271 patients) respectively (OR 1.17, 95% CI 0.75–1.83) [6]. The upper and lower confidence intervals for treatment success in community DOT were 89% and 81% while that of the patients in health facility DOT were 87% and 79%. For smear positive patients the cure rates were 78% for community based patients and 79% for health facility based patients. Given, HIV prevalence among TB patients in Tanzania of 44% [19], and using the formula to calculate cure rate without treatment, it is estimated that, about 11.2% of patients will be cured without TB treatment. When taking into consideration the 'no treatment' scenario, the treatment successes are 74% and 72% for community and health facility based DOT patients respectively.

### Cost-effectiveness

**Figure 4:**
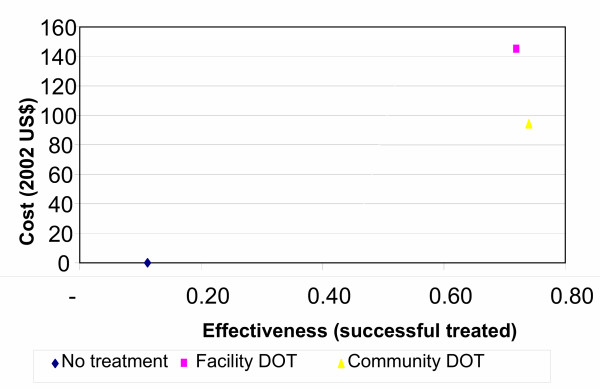
Average cost per patient successfully treated under alternative DOT options.

Figure 4, shows cost-effectiveness of patients treated under community DOT and health facility based DOT. Community based DOT was more cost effective with USD 128 per patient successfully treated, compared to USD 203 for patient successfully treated with health facility DOT. The implementation of community based DOT has improved cost-effectiveness by 37%. For smear positive patients, Community based DOT was cost effective with USD 145 per patient cured while health facility was USD 258 per patient cured. Community based DOT improved cost effectiveness by 44% among smear positive patients.

### Sensitivity analysis

The sensitivity analysis revealed that changes in key input variables did not change the cost-effectiveness ratio of community based DOT in favour of health facility based DOT. Community based DOT remained less costly and more cost-effective (Table 3)

## Discussion

The key finding of this study was that community based DOT was less costly and more cost effective compared to health facility based DOT. This was true from all perspectives: provider's, patients' and treatment supervisors'. Community based DOT maintained programme effectiveness and improved cost-effectiveness by 37%. Implementation of community based DOT reduced cost of treating a patient by one third. This implies that with same level of resources, more TB patients can be successfully treated.

The main reason for the substantial reduction of cost under community DOT was fewer number of visits to the TB clinic. A smear positive patient under health facility DOT made a total of 58 visits to a health facility while a smear negative and extra-pulmonary patient had a total of 24 visits. On the other hand, a patient under community DOT made a total of 10 visits.

The study has a number of limitations, emanating from methodological and operational issues. First, since the study aimed at determining the costs and cost effectiveness of DOT options regardless of the smear status, we combined treatment outcomes and cost data of smear positive, smear negative and extra pulmonary TB patients. In routine practice, the treatment outcomes are reported separately. This may have disproportionately underestimated the cost of treating a smear positive patient under health facility compared to community DOT because, smear negative and extra pulmonary patients under health facility DOT had fewer visits to the TB clinic than smear positives. On the other hand all patients under community DOT had the same number of visits. However, this could not have changed the conclusion of the study since community based DOT remained the less costly option even after these assumptions were tested in sensitivity analysis. Furthermore, patients' treatment outcome and cost data were analysed separately according to TB type and presented as weighted average in the final analysis.

Second, the analysis focussed only on the direct benefit of TB treatment to the individual being treated. We calculated neither the secondary benefit of TB treatment accrued by reduction of transmission in the community nor indirect benefit of community based treatment to the community. Treatment of TB has been shown to have secondary benefit by averting three deaths for every case of TB cured [20]. By considering the benefit of treatment only to the index case, our estimates of the benefit of TB are conservative. However, quantifying the secondary benefits of TB treatment involves complex assumptions, and the inclusion of smear negative and extra pulmonary TB patients would have made such calculations more difficult. Moreover, since TB treatment is already considered an important health intervention [21], and implemented throughout Tanzania, our main interest was to determine cost-effectiveness of alternative TB treatment delivery options.

Finally, health facility cost information was obtained from only one out of five health facilities studied. However, patients and treatment supervisors data were collected from all five-health facilities. The chosen health facility was viewed as broadly representative of the other health facilities in the district providing TB services. Furthermore the health facility had good record keeping of budget and financial details.

Indirect cost in the study has been estimated from time lost due to a visit to TB clinic. There is no agreement among experts on proper way of measuring and valuing indirect cost due to lost time [12-14,22]. We used average reported income among TB patients as baseline value of indirect cost due to lost time. The disadvantage of this method is that it may overestimate or underestimate costs [13]. However, sensitivity analysis was conducted when indirect cost due to lost time was valued to zero and when indirect cost was valued among people with income only. This did not change cost-effectiveness ratio in favour of alternative option.

The main strength of the study is that costing was conducted prospectively alongside a randomised-controlled trial. Quantification and analysis of cost and effectiveness information was carried out in real time and the two groups in the alternative options were comparable.

Although TB services in Tanzania are provided free of charge to the patients, the study showed that patients incurred considerable cost to follow their treatment at the health facility. Patients cost were 30% of the total cost, amounting to $ 43. This is equivalent to their monthly average reported income. These costs did not include expenses used for seeking diagnosis, which have been shown by other studies to be substantial [23,24]. Worth noting also is the fact that indirect costs contributed to about 49% of the total patient's cost.

The findings that community DOT is less costly and more cost effective both from providers and community perspective is consistent with studies conducted elsewhere [25]. Community DOT reduced cost in our study by 35%. Studies in Malawi and Kenya showed that community DOT reduced cost by 50% and 65% respectively [26,27]. Cost reduction was much higher in these settings than in our study. The main reason given is reduction in hospital stay. This is not surprising since we did not include hospitalised TB patients in our study. In Tanzania TB patients are treated on ambulatory basis and admitted only when they are seriously sick.

In order to implement community based DOT, additional cost for initial investments has to be incurred. These include cost for activities such as community supervision and management. This constituted about 53% of providers cost under the community based DOT option. The increased investment, which is minor in absolute term, is nevertheless offset in the long run by savings accrued by implementation of community DOT.

Community based DOT has the potential of increasing the number of TB patients treated without significantly increase in resources. With annual reported TB cases of about 4000, it can plausibly assumed that 2000 more TB patients could be successfully treated in Temeke district, using the current level of resources. This might have wider policy implications in Tanzania and beyond. If community DOT is less costly and is not in any way inferior to conventional health facility treatment, the question is how relevant are these results in other areas beyond the study area. Temeke district represents the growing challenge of controlling TB in urban settings where the numbers of TB patients are increasing and the health system is over-stretched. Our findings should be broadly generalisable in similar settings, since the cost of main items such as staff and drugs are fairly standard. The health facility costs are generally comparable across the country as shown in previous studies [4,15]. The main determinants of patients' cost are not different in many urban centres. The extent to which these findings can be generalised to rural areas is debatable. Evidence suggests that community based DOT is effective even in these settings [7]. Whether it is cost-effective as well is uncertain. The main factors for patients cost; low-income levels and greater distance to health facilities are different from urban settings. Therefore, potential difficulties with widespread implementation of this model must not be underestimated.

## Conclusion

Community DOT presents an economically attractive option to complement health facility based DOT. It improves the affordability and cost-effectiveness of TB treatment. Community DOT might also help to reduce congestion in TB clinics, which are increasingly overburdened by the rising number of patients. This is particularly important in urban centres where TB clinics are working beyond capacity. TB programmes need to consider community DOT as an economically sound and viable option and implement it in the framework of routine programme activities.

## Competing interests

The author(s) declare that they have no competing interests.

## Authors' contributions

All authors participated in designing of the study. EW supervised data collection. EW, OM and BR participated in data analysis, writing and editing of the paper. All authors read and approved the final manuscript.
